# Expression of thromboxane synthase, TBXAS1 and the thromboxane A2 receptor, TBXA2R, in human breast cancer

**DOI:** 10.1186/1477-7800-2-23

**Published:** 2005-10-26

**Authors:** Gareth Watkins, Anthony Douglas-Jones, Robert E Mansel, Wen G Jiang

**Affiliations:** 1 Metastasis & Angiogenesis Research Group, Wales College of Medicine, Cardiff University Cardiff UK

## Abstract

**Background:**

Thromboxane synthase (TxS) metabolizes the cyclooxygenase product, prostaglandin H(2), into thromboxanes. Some of the thromboxanes are known to be biologically active on cancer cells. The aim of the study was to investigate the expression of thromboxane synthases, TBXAS1 and the thromboxane A2 receptor, TBXA2R in a cohort of human breast cancer patients and also to assess their potential clinical relevance.

**Methods:**

Human breast tumour tissues (n = 120) and non-neoplastic mammary tissues (n = 32) were studied. Levels of TBXA2R and TBXAS1 transcripts were quantified using quantitative real-time RT-PCR analysis and correlated with clinical/pathological information including nodal status, grade, prognosis and long term survival (median follow-up period 120 months).

**Results:**

Breast tumour tissue expressed higher levels of TBXA2R compared with normal mammary tissues, although the difference was not statistically significant (p = 0.09). There was no difference between tumour and normal tissues for TBXAS1. However, TBXA2R expression was significantly increased in grade 3 tumours(p = 0.006 vs grade 1), while TBXAS1 was significantly reduced in grade 3 tumours (p = 0.026 vs grade 1 tumours). A similar differential expression pattern was seen in tumours from patients with different prognosis, in that patients with predicted poor prognosis had higher, but not statistically different, levels of TBXA2R, and significantly lower levels of TBXAS1 (p = 0.008). Finally, Kaplan-Meier survival analysis has shown that patients with high levels of TBXA2R had significantly shorter disease free survival (103.8 (79.1–128.5) months) compared with those with low levels (123.7 (112.0–135.3)) months, p = 0.043.

**Conclusion:**

Thromboxane synthases are differentially expressed in human breast cancer. While TBXA2R is highly expressed in aggressive tumours and linked with poor prognosis, TBXAS1 is expressed at significantly low levels in high grade tumours and tumour patients with poor prognosis. TBXA2R thus has a significant prognostic value in clinical breast cancer.

## Background

Prostaglandin metabolites (eicosanoids) are known to be selectively active in regulating functions in cells including cancer cells [1,2]. For example, 12-HETE and 13-HODE have been shown to act as pro- and anti-cancer eicosanoids in a range of cancer cells. Eicosanoids are generated from prostaglandins by specific enzymes, some of which have been shown to be actively involved in the development and progression of cancers [3,4]. We have previously reported aberrant expression of the other groups of prostaglandin enzymes 5-, 12-, 15 LOX and COX-2 in human breast cancer and have demonstrated a distinct pattern of difference with these enzymes [5].

Thromboxane synthase (TxS) metabolizes the cyclooxygenase product, prostanglandin H(2), into thromboxane A(2) (TXA(2)), which can cause vessel constriction, platelet activation, and aggregation. In human prostate cancer, thromboxane synthase has been found to be weakly expressed or absent in normal differentiated luminal or secretory cells, significantly elevated in less differentiated or advanced prostate tumors, and markedly increased in tumors with perineural invasion [6]. Over-expression of the enzyme in prostate cancer cells increased the cellular motility [6]. The same over expression of TBXA2R has been seen in adenocarcinoma and squamous cell carcinoma of the lung [7].

Thromboxane synthase inhibitors have been shown to induce apoptosis in glioma cells [8]. In a colorectal tumour model, cancer cells transduced with TXA(2) synthase cDNA produced faster growing tumours, an effect that can be reversed by TXA inhibitors [9].

In cancer cells, including breast cancer cells, TBXA2R has been shown not to influence the adhesion of cancer cells to matrix proteins [10]. Interestingly, TBXA2R has been shown to influence angiogenesis in a lung tumour model, potentially by affecting the migration of endothelial cells [11]. Thromboxane synthase has been shown to be associated with metastasis of renal cell carcinoma [12]. Increased expression of TXA synthase enzymes is a feature of differentiated monocytoid leukaemia cell lines [13]. Early studies using thromboxane synthase inhibitors in vivo have failed to show any beneficial effects on metastasis or spread to lymph nodes [14]. Thromboxane (TX) synthase inhibitors and a TBXA2R receptor antagonist have been found to inhibit the formation of metastasis from tail vein injected B16a cells, as well as reduce spontaneous metastasis from subcutaneous B16a and Lewis lung carcinoma tumours [15].

In the current study, we have investigated the level of expression of Thromboxane A synthase 1, TBXAS1 (otherwise known as Cyp5 and TXS) and Thromboxane A2 receptor, TBXA2R, in a cohort of human breast cancer patients. In addition, we have analysed the clinical and prognostic relevance of both enzymes with the clinical outcome of the patients over a 10 year period.

## Materials and methods

### Tissues and patients

The cohort of breast tissues and patients were as previously described [5,16], except that the median follow-up for the patients was 120 months. 120 tumour tissues and 32 normal tissues were included in the current study, in which normal tissues were obtained from the same patients with breast tumour but were away from tumour margins. The normal background tissues were free from tumour involvement as confirmed by histological examination. The pathological data, tumour staging, histological types and the initial prognostic index for the patients were previously described and is given in table 1.

**Table 1:** Clinical and pathological details of the study cohort

	**Negative (n =)**	**Positive (n =)**			
**Nodal status n =**	65	55			
**ER status**	71	49			
					
**Grade**	**Grade 1**	**Grade 2**	**Grade 3**		

n =	23	41	56		
					
			**Others**		
			
**Histology**	**Ductal**	**Lobular**	**medullary**	**tubular**	**mucinous**

n =	88	14	2	2	4
					
**TNM staging**	**TNM 1**	**TNM 2**	**TNM 3**	**TNM 4**	

**n =**	69	40	7	4	
					
**Clinical outcome**	**Disease free**	**With Metastasis**	**With local recur.**	**Died of breast Cancer**	**Died of unrelated diseases**

n =	81	7	5	20	7

### Tissue processing and extraction of RNA and generation of cDNA

Tissue processing was as we previously reported [17]. Frozen tissues were sectioned using a cryostat and stored at -20°C until use. Over 20 frozen sections from the tissues were homogenised in a RNA extraction solution using a hand held homogeniser to extract total RNA. The concentrations of RNA were quantified using a UV spectrophotometer. 1 μg RNA was used to generate cDNA using a commercially available RT kit (AbGene Laboratories, Essex, England, UK).

### Quantitative analysis of thromboxane synthases

The level of thromboxane synthases transcripts from the above prepared cDNA was determined using a real-time quantitative PCR, based on the Amplifluor technology [18-20]. Briefly, Primers were designed using the Beacon Designer software (version 2, Palo Alto, California, USA), to amplify regions of human thromboxane synthases that have no significant overlap with other known sequences and that the amplified products span over at least one intron. Primers for TBXA2R (gene bank accession number U27325) were 5'tcagctcctggggatcat'3 and 5'actgaacctgaccgtacaggtttcgcagcactgtct'3 and for TXA synthase (gene bank accession number NM_030984) 5'caggtgttggttgagaactt'3 and 5'actgaacctgaccgtacatgtcacgtaaaaacagaacg'3. To one of the primer was added an additional sequence, known as the Z sequence (5'actgaacctgaccgtaca'3) which is complementary to the universal Z probe (Intergen Inc., Oxford, England, UK). A Taqman detection kit for β-actin was purchased from Perkin-Elmer. The reaction was carried out using the following: Hot-start Q-master mix (Abgene), 10 pmol of specific forward primer, 1 pmol reverse primer which has the Z sequence, 10 pmol of FAM-tagged probe (Intergen Inc.,), and cDNA from approximate 50 ng RNA. The reaction of was carried out using a IcyclerIQ (Bio-Rad) which equipment with a optic unit that allows an real time detection of 96 reactions, using the following condition: 94°C for 12 minutes, 50 cycles of 94°C for 15 seconds, 55°C for 40 seconds and 72°C for 20 seconds. The levels of the transcripts were generated from a standard that were run together with the samples.

Statistical analysis was carried out using Mann-Whitney U test and the Kruskal-Wallis test and survival analysis using Kaplan-Meier survival curve and Univariate analysis (SPSS12).

## Results

### Mammary tissues differentially expressed both TBXA2R and TBXAS1

**Figure 1:**
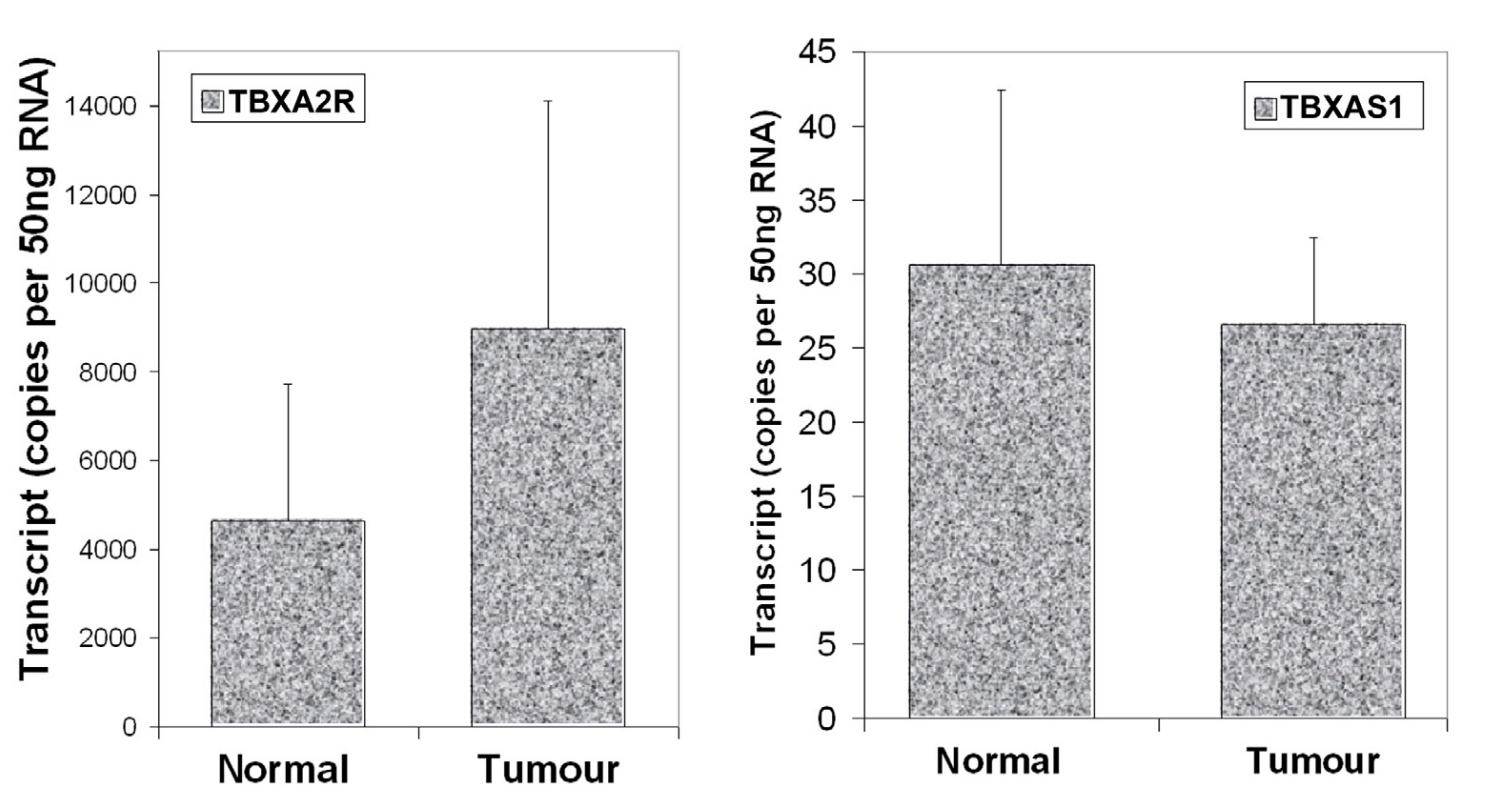
Levels of TBXA2R (left) and TBXAS1 (right) in mammary tissues.

Breast tissues expressed both TBXA2R and TBXAS1, although levels of TBXA2R transcripts tended to be higher than that of TBXAS1 (figure 1). When normal mammary tissues and tumour tissues were compared, TBXA2R was found to be higher in tumour tissues than in normal tissues, although this is not statistically significant (p = 0.09). The levels of TBXAS1 transcript were identical between normal and tumour tissues (figure 1 left).

### TBXA2R and TBXAS1 transcript levels are differentially linked to tumour differentiation

**Figure 2:**
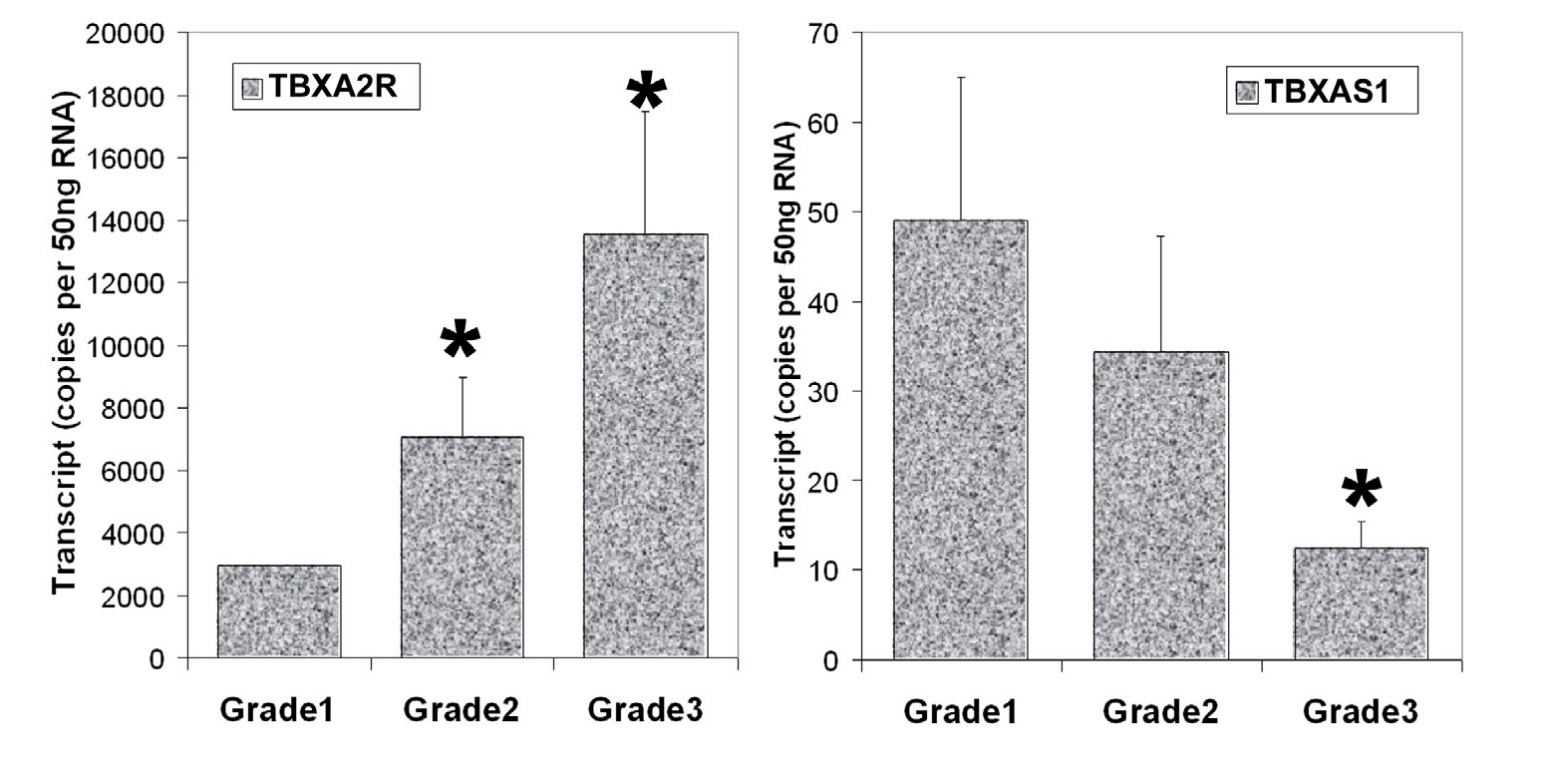
Levels of TBXA2R (left) and TBXAS1 (right) and their relationship with tumour grade. * p < 0.05 vs grade 1.

In the current study, we have observed that there was significant correlation between levels of TBXA2R and TBXAS1 and tumour grade. As revealed in figure 2 (left), grade 2 and grade 3 tumours had significantly higher levels of TBXA2R transcript (p = 0.0158 and p = 0.006 vs grade 1, respectively). Grade 3 tumour had significantly lower levels of TBXAS1 compared with grade 1 tumours (p = 0.026). The difference between grade 1 and grade 2 was not significant.

When levels were compared between node positive and node negative tumours, no significant difference was seen with either enzyme (5189 ± 2244 vs 13222 ± 10821 p = 0.48, and 33.2 ± 10 vs 18.5 ± 6.3, p = 0.21, for TBXA2R and TBXAS1, respectively).

The levels of both molecules were also analysed against ER status, based on a recent study [21]. As shown in table 2, ER positive tumours had significantly higher levels of TBXA2R compared with ER negative tumours (p=0.0128). No significant difference was seen with TBXAS1 in different ER status. We also compared the two major histological types of breast tumours in the current study, namely ductal and lobular tumours. As shown in table 3, no significant difference was seen between different types.

**Table 2:** TBXA2R and TBXAS1 levels and ER status

	ER (-)	ER (+)	P value
TBXA2R	2286 ± 1402	21549 ± 16583	**p = 0.0128**
TBXAS1	16.4 ± 3.6	46.9 ± 18.5	p = 0.12

**Table 3:** TBXA2R and TBXAS1 levels and relationship with histological types

	Ductal	Lobular	P value
TBXA2R	9363 ± 6542	5541 ± 5337	p = 0.67
TBXAS1	23.1 ± 6.1	22.8 ± 13.9	p = 0.34

### Thromboxane synthase levels, prognosis and clinical outcomes

In the current study, we have used two methods in assessing the prognosis and clinical outcome. The first method was using a predictive indicator, the Nottingham Prognostic Index (NPI). Patients were divided into three different prognostic groups, those with predicted good prognosis (NPI1<3.4), moderate (NPI2 3.4–5.4) and poor prognosis (NPI3>5.4). As shown in table 1, patients with predicted poor prognosis had high levels of TBXA2R, however, the difference was not significant. It is noteworthy that the levels of TBXAS1 transcript were significantly lower in patients with predicted poor prognosis (table 4).

**Table 4:** TBXA2R and TBXAS1 levels and prognosis.

	Good	Moderate	Poor
TBXA2R	5188 ± 2244	2561 ± 1206 (p = 0.30)	37614 ± 35470 (p = 0.38)
TBXAS1	33.2 ± 10	20.8 ± 8.2 (p = 0.086)	**12.1 ± 6.7 (p = 0.008)**

P values are comparison with patients with good prognosis.

The second method was to compare levels of the enzyme transcript based on the clinical outcomes of the patients, over a 10 year follow-up. This resulted in a sub division of patients into two groups:- those who remained disease free or who developed metastasis or local recurrence, and those who died of breast cancer (excluding non-cancer related deaths). As shown in table 5, although a high level of TBXA2R was seen in patients who died of breast cancer, this was not significant. No significant differences in TBXA2R and TBXAS1 transcript were seen between groups with difference clinical outcomes.

**Table 5:** TBXA2R and TBXAS1 levels and the clinical outcome.

	Disease free	With metastasis	With local recurrence	Patients who died of breast cancer
TBXA2R	3405 ± 1503	8660 ± 3661 (p = 0.11)	12882 ± 12873 (p = 0.52)	38611 ± 32930 (p = 0.40)
TBXAS1	22.3 ± 5.5	10.6 ± 4.3 (p = 0.10)	48 ± 47 (p = 0.62)	25.7 ± 12.4 (p = 0.81)

P values are comparison with patients who remained disease free.

### Thromboxane synthase and long term survival

**Figure 3:**
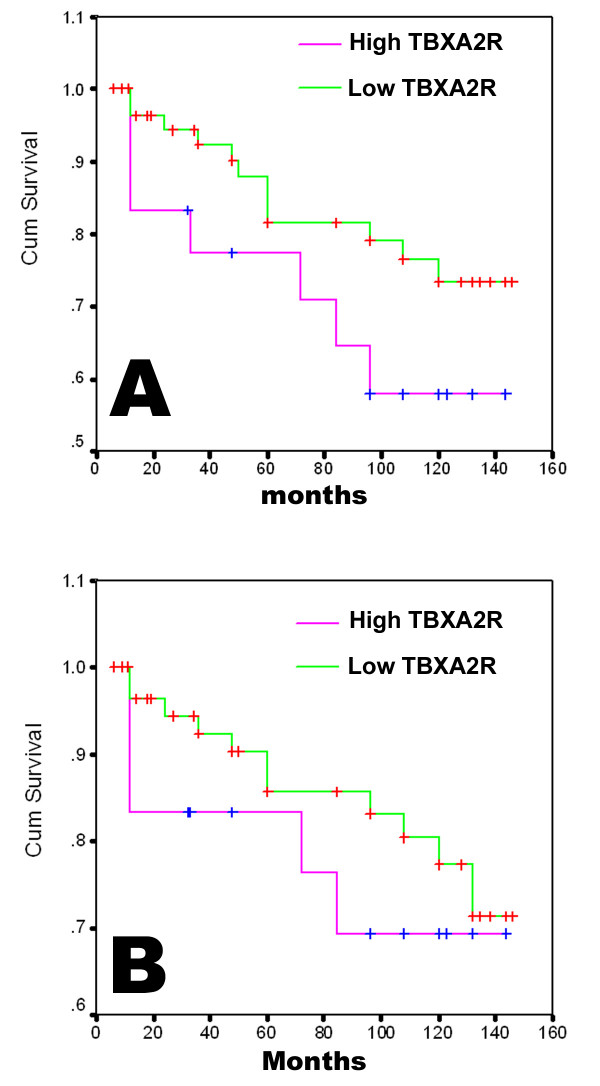
TBXA2R and long term disease free (A) and overall (B) survival. High TBXA2R levels were associated with significant shorter disease free survival (A), p = 0.043.

The value of TBXA2R and TBXAS1 in assessing long term survival was analysed using the Kaplan-Meier survival and Univariate analysis. As seen in figure 3A, high levels of TBXA2R transcript was associated with a significantly shorter disease free survival (103.8 (79.1–128.5) months vs 123.7 (112.0–135.3) month, for patients with high and low levels, respectively, p = 0.043). There was no significant correlation between TBXA2R and the overall survival (figure 3B), 111.8 (84–139.3) vs 126.1 (115.5–136.7) months, for patients with high and low TBXA2R, p = 0.43, figure 3B). The comparison for the subgroups with different levels of TBXAS1 returned no significant difference for the overall survival (p = 0.43) and disease free survival (p = 0.412).

## Discussion

The current study has reported that in human breast cancer, transcript levels of thromboxane synthases are aberrant. While TBXA2R is normally expressed in tumours and particularly in aggressive tumours, TBXAS1, however, showed a very different expression pattern from that of TBXA2R. Furthermore, TBXA2R levels are associated with disease free survival.

High levels of TBXA2R and TBXA2Rs have been previously reported in various tumours including prostate, glioma, and melanoma. As a result, previous studies have shown that inhibitors/antagonists to TBXA2R are able to inhibit metastatic spread. In a liver metastasis model from colon cancer, the thromboxane synthase inhibitor, sodium ozagrel at a dose of 15 mg/kg body weight, has been shown to significantly reduce the rate of metastatic tumours in the liver, by 57% [22]. Ketoconazole has been shown to act as an antagonist to both thromboxane synthase and 5-LOX [23]. Ketoconazole has been shown to reduce lung metastasis from melanoma cells in an in vivo model. This together with the current study indicates that TBXA2R, has a potential impact, via its metabolised product TAX2, on the metastatic nature of cancer cells.

Metastasis is a complex biological and clinical phenomenon that involves a number of highly independent yet crucial steps during its development. Thromboxane synthase has been shown to influence a number of steps in the process including cell migration, angiogenesis and apoptosis. Certain eicosanoids (i.e. PGE2) may also stimulate oestrogen synthesis and contribute to the growth of breast tumours, via pathways including that of aromatase [24,25]. In addition, eicosanoids also interplay with other lipids, by way of the peroxisome proliferators activated receptors (PPAR) [26] which are known to be aberrantly expressed in breast cancer [19].

The level of expression of thromboxane synthase in glioma cells appears to correlate with the speed of migration [27]. Similarly, TBXA2R expression has been shown to influence endothelial cell migration and angiogenesis [11]. Nevertheless, anomalies exist as some inhibitors to thrombaxane synthase, such as dazmegrel, failed to impact on the growth and weight of fibrosarcoma [28,29].

The other noteworthy observation that can be derived from the current study is the expression pattern of TBXAS1, which is in clear contrast to that of TBXA2R. TBXAS1 is expressed at significantly low levels in high grade tumours (grade 3) and in patients with predicted poor clinical outcome (prognostic index NPI greater than 5.4). This indicates that metabolites generated by TBXAS1 may play a very different role in cancer to that of TBXA2R. There is very little experimental data available on this aspect. Future studies on the TBXAS1 in vitro and in vivo would be of significant interest. Finally, the current study has shown that ER positive tumours have significantly higher TBXA2R levels than ER negative tumours. Given the ongoing interest in COX-2 inhibitors and aromatase inhibitors [30,31], it would be of interest to take into consideration of TBXA2R, ER status as well as aromatase expression profile, when considering trial design and choice of therapies.

A number of factors are known to regulate the expression of TXA synthases. Factors upregulating thromboxane synthase include phorbol ester [34], Activin A [32], Benzoquinone derivatives [33] which are interesting as some of the compounds are dually active on TBXA2R and 5-LOX. Expression of thromboxane synthase is regulated by cis-elements, trans-activators and potentially by genomic methylation [35]. These early observations have an important bearing when considering the current results.

In conclusion, expression of thromboxane synthases in clinical human breast cancer is aberrant. While TBXA2R is commonly expressed in tumours and particularly in aggressive tumours, TBXAS1, in contrast, showed a very different expression pattern from that of TBXA2R. Furthermore, TBXA2R levels are associated with disease free survival, indicating that the receptor is a prognostic factor in clinical breast cancer.

## List of abbreviations

12-HETE: 12-hydroxyeicosatetraenoic acid;

13-HODE: 13- hydroxyoctadecadienoic acid;

COX-2: cyclooxygenase-2;

ER: oestrogen receptor;

LOX: lipoxygenase;

PGE2: prostaglandin E2;

TBXA2R: thromboxane A2 receptor;

TBXAS1: thromboxane A synthase 1;

TXA2: thromboxane A2;

TxS: Thromboxane synthase;

## Competing interests

The author(s) declare that they have no competing interest.

## Authors' contributions

GW: conducting experiments, data analysis and manuscript preparation.

REM: study design, sample collection and MS preparation.

ADJ: Histological analysis.

WEJ: study design, experimental design, data analysis, MS preparation.
